# Hand, Foot, and Mouth Disease Caused by Coxsackievirus A6

**DOI:** 10.3201/eid1810.120813

**Published:** 2012-10

**Authors:** Kelly Flett, Ilan Youngster, Jennifer Huang, Alexander McAdam, Thomas J. Sandora, Marcus Rennick, Sandra Smole, Shannon L. Rogers, W. Allan Nix, M. Steven Oberste, Stephen Gellis, Asim A. Ahmed

**Affiliations:** Children’s Hospital Boston, Boston, Massachusetts, USA (K. Flett, I. Youngster, J. Huang, A. McAdam, T.J. Sandora, S. Gellis, A.A. Ahmed);; Boston Public Health Commission, Boston (M. Rennick);; Massachusetts Department of Public Health, Jamaica Plain, Massachusetts, USA (S. Smole);; and Centers for Disease Control and Prevention, Atlanta, Georgia, USA (S.L. Rogers, W.A. Nix, M.S. Oberste)

**Keywords:** Coxsackievirus A6, hand, foot and mouth disease, enterovirus, viral, exanthema, viruses

**To the Editor:** Coxsackievirus A6 (CVA6) is a human enterovirus associated with herpangina in infants. In the winter of 2012, we evaluated a cluster of 8 patients, 4 months–3 years of age, who were brought for treatment at Boston Children’s Hospital (Boston, MA, USA) with a variant of hand, foot, and mouth disease (HFMD) that has now been linked to CVA6 (Table). During this same period, the Boston Public Health Commission’s syndromic surveillance system detected a 3.3-fold increase in emergency department discharge diagnoses of HFMD. In the United States, HFMD typically occurs in the summer and early autumn and is characterized by a febrile enanthem of oral ulcers and macular or vesicular lesions on the palms and soles; the etiologic agents are most often CVA16 and enterovirus 71.

**Table:** Demographic and clinical characteristics of patients with CVA6-associated HFMD, Boston, Massachusetts, USA, 2012*

Patient no.	Age/sex	Date treatment sought	Tmax, °C	URI	Lesions				Specimen used for CVA6 diagnosis†
					Perioral	Intraoral	Perirectal	Hands/feet	
1	12 mo/M	Feb 15	39.8	No	Yes	No	Yes	Yes	VF
2	10 mo/F	Feb 2	38.2	Yes	Yes	Yes	Yes	Yes	VF
3	8 mo/M	Mar 1	39.1	No	Yes	No	Yes	No	TS/RS
4	2 y/M	Mar 1	38.4	Yes	Yes	Yes	Yes	Yes	TS/RS
5	4 mo/F	Feb 28	38.3	No	Yes	No	No	Yes	VF
6	1.5 y/M	Mar 16	39.4	Yes	Yes	No	Yes	No	TS/RS
7	3 y/M	Mar 8	38.0	Yes	Yes	Yes	Yes	Yes	NP
8	15 mo/M	Mar 7	39.5	No	Yes	Yes	Yes	Yes	NP/RS

*CVA6, coxsackievirus A6; HFMD, hand, foot, and mouth disease; Tmax, maximum measured temperature; URI, upper respiratory infection, including symptoms of congestion or cough; RT-PCR, reverse transcription PCR; +, positive; VF, vesicular fluid; TS, throat swab; RS, rectal swab; NP, nasopharyngeal swab. †Diagnosis was made by sequencing and analysis of the viral protein 1 coding region after RT-PCR.

In contrast to the typical manifestation, the patients in the Boston cluster exhibited symptoms in late winter (Table) and had perioral (Figure, panel A) and perirectal (Figure, panel B) papules and vesicles on the dorsal aspects of the hands and feet (Figure, panel C). Patients experienced a prodrome lasting 1–3 days, consisting of fever (8 patients), upper respiratory tract symptoms (4 patients), and irritability (7 patients). This prodrome was followed by the development of a perioral papular rash (8 patients), which was often impetiginized with secondary crusting; a prominent papulovesicular rash on the dorsum of the hands and feet (6 patients); and a perirectal eruption (7 patients). Half of the patients had intraoral lesions. Fever abated in most of the patients within a day after onset of the exanthem. The rash resolved over 7–14 days with no residual scarring. Samples from the oropharynx, rectum, and vesicles from these patients were sent to the Centers for Disease Control and Prevention (Atlanta, GA, USA) for analysis. Reverse transcription PCR and sequencing by using primers specific for a portion of the viral protein 1 coding region identified CVA6 (*1*) (Table).

**Figure:**
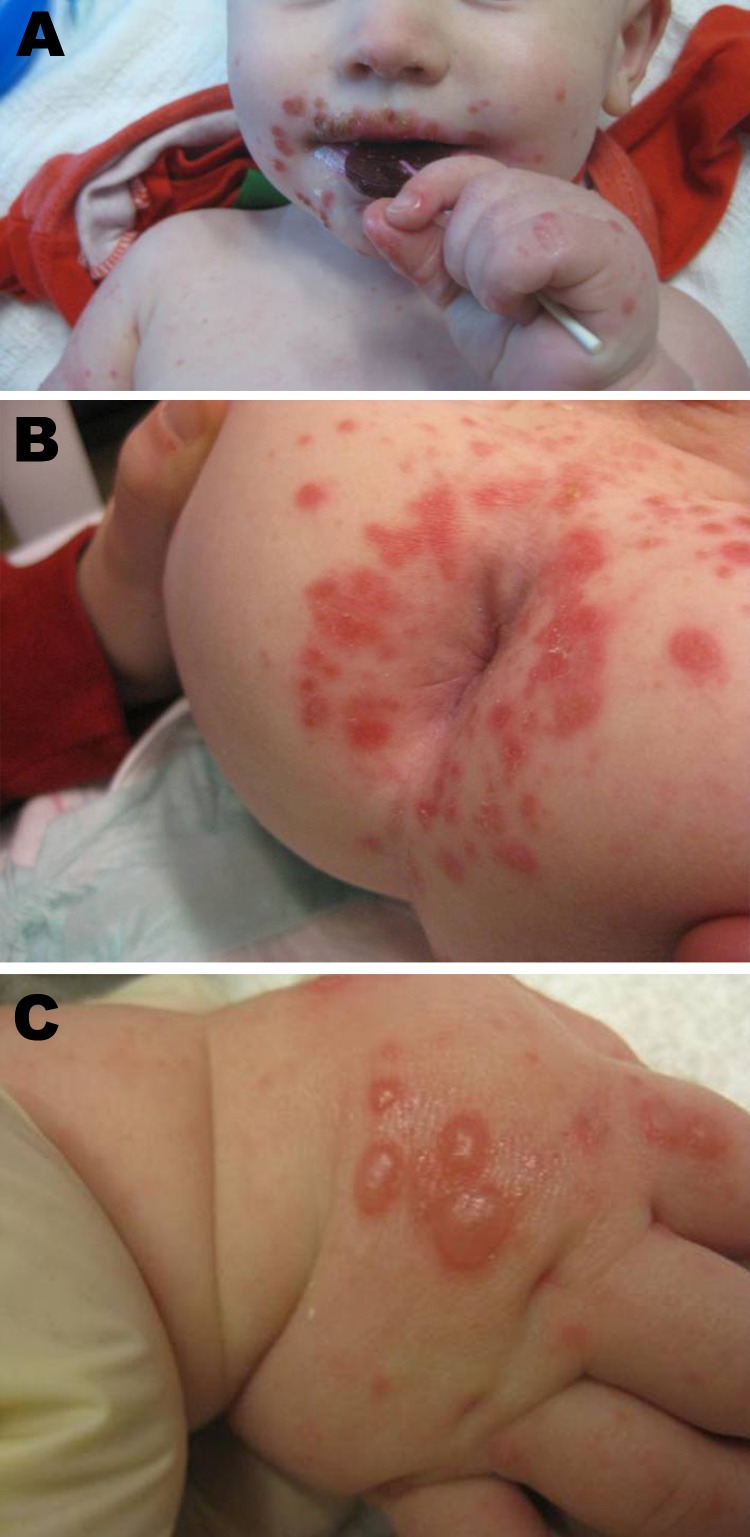
Manifestations of hand, foot, and mouth disease in patients, Boston, Massachusetts, USA, 2012. Discrete superficial crusted erosions and vesicles symmetrically distributed in the perioral region (A), in the perianal region (B), and on the dorsum of the hands (C).

Outbreaks of HFMD caused by CVA6 have been described in Singapore, Finland, Taiwan, and most recently in Japan; most cases have occurred in the warmer months (*2*–*6*). Cases in the cluster described here are likely related to an emerging outbreak of CVA6-associated HFMD in the United States (*7*). The atypical seasonality of the outbreak, during the winter in Boston, could be related to the unusually mild temperatures in the winter of 2012.

Recent CVA6 outbreaks have been characterized by a febrile illness associated with an oral enanthem and lesions on the palms, soles, and buttocks. CVA6 infections in Taiwan during 2004–2009 were associated with HFMD in 13% of cases, with disease defined as oral ulcers on the tongue or buccal mucosa and vesicular rashes on the palms, soles, knees, or buttocks (*2*). In Singapore, where CVA6 accounted for 24% of HFMD cases, patients had oral lesions and <5 peripheral papules, placing them on a spectrum closer to the herpangina more typically observed in CVA6 infection (*8*).

The patients we report in this cluster most typically had perioral and perirectal papules in addition to vesicles on the dorsum of their hands. Two reports of CVA6-associated HFMD outbreaks describe cases that more closely resemble patients in the Boston outbreak. In a series from Finland in 2008, representative patients had both perioral lesions and vesicles on the dorsum of their hands (*6*). In a large series of patients with HFMD in Taiwan in 2010, patients with CVA6 had perioral lesions in addition to an enanthem (*3*).

Outbreaks of CVA6-associated HFMD in Finland, Taiwan, and Japan were associated with onychomadesis, with the loss of nails occurring 1–2 months after initial symptoms (*3*,*4*,*6*). The association between more typical HFMD and onychomadesis has additionally been described in the United States and Europe but without a link to specific serotype or with a small percentage of CVA6-associated cases (*9*). Cases from the Boston epidemic may fit into an emerging clinical phenotype of CVA6, and it will be interesting to see whether nail loss develops in those patients.

Given the numerous CVA6 outbreaks in multiple countries in 2008 and a US population that may be relatively naïve to this serotype, CVA6 is likely to spread throughout North America. Clinicians should be aware that, although standard precautions are routinely recommended for managing enteroviral infections in health care settings, contact precautions are indicated for children in diapers to control institutional outbreaks (*10*). In addition, the presence of perioral lesions and peripheral vesicles on the dorsum rather than palmar/plantar surface of the hands and feet represents a unique phenotype of HFMD that could be confused with herpes simplex or varicella-zoster virus infections. Because of the atypical presentation of CVA6-associated HFMD, clinical vigilance is needed to recognize emerging regional outbreaks. More detailed epidemiologic and genetic analyses will be required to characterize the role of CVA6 in US outbreaks of HFMD.
